# Conservative Management of a Biliary Tract Injury During Percutaneous Nephrolithotomy

**DOI:** 10.7759/cureus.50697

**Published:** 2023-12-17

**Authors:** Raghubir Bhardwaj, Abhik Chatterjee, Ratnadip Deb

**Affiliations:** 1 Urology, Tata Main Hospital, Jamshedpur, IND; 2 Surgery, Manipal Tata Medical College, Tata Main Hospital, Jamshedpur, IND

**Keywords:** complications, conservative management, biliary tract, percutaneous nephrolithotomy (pcnl), staghorn stone

## Abstract

Percutaneous nephrolithotomy (PCNL) is a well-established treatment option for the management of kidney stones. It has evolved over time with advances in surgical technique and technology. Even for the most experienced urologists, both major and minor complications may be encountered during PCNL. Timely diagnosis and appropriate management are the keys to a favourable outcome. Here, we discuss the case of a woman aged 34 years, who underwent PCNL for a right renal staghorn stone and had an accidental puncture of the gall bladder. Post-operatively, the patient recovered well with conservative management.

## Introduction

Percutaneous nephrolithotomy (PCNL) has established itself as a safe and routinely used technique for the treatment of renal calculi [1]. The number of PCNL procedures is increasing worldwide as patients with complex renal calculi and comorbidities are being treated with this minimally invasive technique [2]. Like any other surgical procedure, PCNL is not without complications. Major complications can occur in 7% of patients and minor complications in 25% of patients. Commonly encountered complications are fever (21-32%), bleeding (10-18%), thoracic complications (<2%), urosepsis (1-2%), colonic injury (0.2-0.8%), and solid organ injuries [3]. Biliary tract injury is a rare but serious complication of PCNL [4]. We present a case of biliary tract injury during PCNL and its conservative management.

## Case presentation

**Figure 1:**
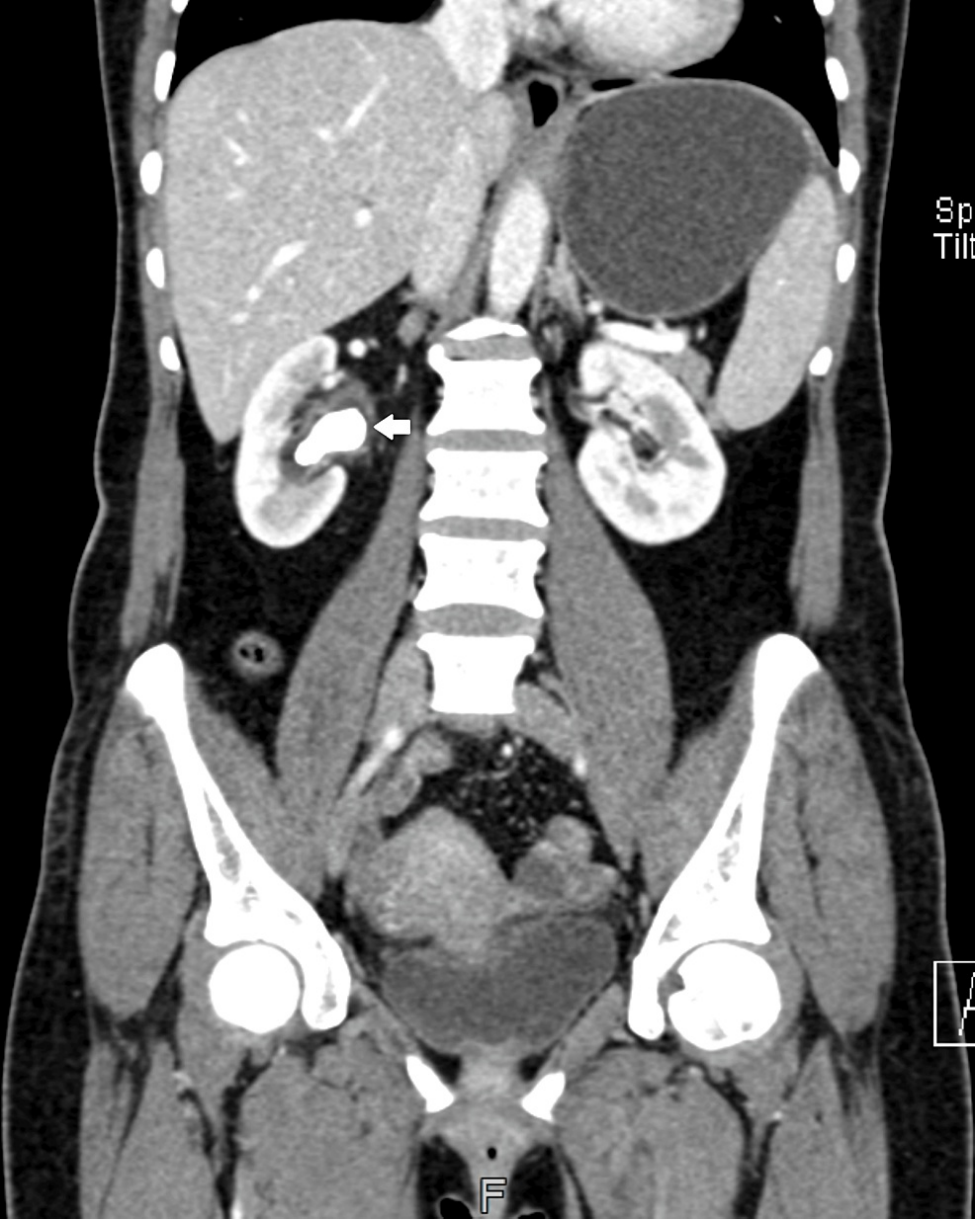
Contrast-enhanced computed tomography scan shows the right renal stone.

A 34-year-old moderately built patient with a body mass index (BMI) of 24.7 kg/m^2^ was admitted to our hospital with a history of symptomatic right staghorn stone. A percutaneous nephrolithotomy was planned. Preoperative workup showed serum creatinine of 0.85 mg/dl and hemoglobin of 10.5 g/dl. Urine culture showed no growth. There is no history of diabetes mellitus or hypertension. The patient gave a history of uneventful lower-segment cesarean section in 2012. Ultrasound showed a staghorn stone of 2.5 cm x 2.2 cm size in the right renal pelvis. CT urography showed a large staghorn stone measuring 2.8 cm x 2.2 cm x 1.2 cm in the right renal pelvis with moderate hydronephrosis (Figure 1).

The right ureter was normal in course and calibre. A pre-anesthetic check-up was done, and the patient was cleared for the surgery.

The procedure was performed under general anesthesia. Cystoscopy, right pyelogram, and right ureteric catheterization were done. The position was changed from lithotomy to prone. The lower calyx was punctured under C-arm guidance, and the tract was serially dilated. The stone was seen, and fragmentation started. During fragmentation, a part of the stone migrated into the upper calyx. It was decided to make a puncture through the upper calyx for the clearance of the migrated stone. As we punctured the upper calyx under C-arm guidance and removed the stylet of the needle, the bile started coming out of the needle. The procedure was stopped in view of the suspected biliary tract injury. A 6F double-J stent and nephrostomy tube were placed through the lower calyx tract. A nasogastric tube was inserted at the end of the procedure, and the patient was shifted to the high-dependency unit for careful monitoring.

A surgical gastroenterologist’s opinion was taken. The patient was started on IV antibiotics and monitored with parameters, such as abdominal girth, abdominal palpation for tenderness, abdominal percussion for any change in the percussion note, blood pressure, pulse rate, temperature, respiratory rate, nasogastric tube aspirate, urine output, and urine color. Postoperative blood tests showed serum creatinine of 0.7 mg/dl and hemoglobin of 8.5 g/dl. Mild abdominal distension was observed for 20-24 hours, with no history of vomiting.

Ultrasound was performed 48 hours after the surgery, which showed a collapsed gallbladder with collection in the pelvis. Liver function tests showed high total and direct bilirubin. Contrast-enhanced computed tomography (CT) scan abdomen was done 72 hours after the surgery. It showed a complete resolution of the pelvic collection. Clinical examination on the fourth postoperative day showed a soft, nontender abdomen; hence, oral feeding was started. Total and direct bilirubin were within normal limits on postoperative day five. The patient was discharged on the seventh postoperative day. A follow-up visit after two weeks revealed normal creatinine and bilirubin. Follow-up visits at one, three, and six months showed an uneventful recovery and no history of abdominal pain or fever. Serum bilirubin and serum creatinine were within normal limits.

## Discussion

PCNL is an effective treatment for staghorn renal stones but not without the risk of complications that may endanger life, like any other surgical procedures [5]. Fever is the most common complication after PCNL. Major complications, such as renal pseudoaneurysm, which may require angioembolization and colonic and pleural injuries, constitute about 3.2-6.8%. Gall bladder puncture or biliary tract injury is one of the uncommon complications of PCNL but with potentially grave consequences [6]. A well-distended gall bladder is in the close relation to the right kidney and may get injured, especially when percutaneous access is on the medial side. In the literature, there are only few documented cases of gall bladder injury during PCNL. Most of the reported cases have undergone exploratory laparotomy and cholecystectomy [7].

Abdominal pain, tenderness, abdominal distension, vomiting, diarrhea, fever, and tachycardia are some of the important signs and symptoms of biliary peritonitis [8]. Early diagnosis and timely management are the key to the uneventful outcome. Delay in the diagnosis and further management can increase mortality secondary to biliary peritonitis. Imaging modalities in the form of contrast-enhanced CT scan of the abdomen can help to clinch the early diagnosis. Magnetic resonance cholangiopancreatography (MRCP) is an important investigation when an injury to the biliary tract is suspected [9]. When damage to the gall bladder is confirmed with the imaging modality, in most cases, cholecystectomy is the treatment of choice. However, when a biliary tract injury is confirmed, minimally invasive procedures, such as endoscopic retrograde cholangiopancreatography (ERCP) is an important treatment modality.

In most of the cases of biliary tract injuries, diagnosis gets delayed until the patient develops symptoms of peritonitis. In these cases, conservative treatment is not advisable, and immediate exploratory laparotomy is the best line of management [10]. Delay in diagnosis and treatment may increase mortality significantly. Late complications of biliary tract injuries include biliary strictures, recurrent cholangitis, and biliary cirrhosis, which signifies the importance of a careful follow-up after discharge. 

In our case, the patient was managed conservatively. During conservative management, the patient is closely monitored using clinical, biochemical, and radiological modalities. If there is deterioration in the patient’s condition, immediate cholecystectomy is mandatory to reduce the risk to the patient’s life.

## Conclusions

Gall bladder injury is a rare complication of PCNL. A high degree of clinical suspicion is essential to the early diagnosis and prompt management. Radiological evaluation helps to confirm the diagnosis especially when a clinical presentation is not conclusive. Most of the cases need surgical intervention especially when the diagnosis gets delayed. Conservative management that needs careful monitoring is an option in otherwise stable cases.
